# Changes in Phlebotomine Sand Fly Species Composition Following Insecticide Thermal Fogging in a Rural Setting of Western Panamá

**DOI:** 10.1371/journal.pone.0053289

**Published:** 2013-01-09

**Authors:** Jose E. Calzada, Azael Saldaña, Chystrie Rigg, Anayansi Valderrama, Luz Romero, Luis Fernando Chaves

**Affiliations:** 1 Departamento de Parasitología, Instituto Conmemorativo Gorgas de Estudios de la Salud, Ciudad de Panamá, Panamá; 2 Departamento de Entomología, Instituto Conmemorativo Gorgas de Estudios de la Salud, Ciudad de Panamá, Panamá; 3 Programa de Investigación en Enfermedades Tropicales, Universidad Nacional, Heredia, Costa Rica; 4 Graduate School of Environmental Sciences, Hokkaido University, Sapporo, Japan; University of California, Riverside, United States of America

## Abstract

American Cutaneous Leishmaniasis, ACL, is a zoonotic disease with a large richness of co-occurring vector species in transmission foci. Here, we describe changes in patterns of phlebotomine sand fly (Diptera: Psychodidae: Phlebotominae) species composition at the village of Trinidad de Las Minas, Capira, Panamá, a hyperendemic focus of ACL transmission, subjected to a vector control intervention with insecticide thermal fogging (ITF). Our study setting consisted of 24 houses, 12 subjected to two rounds of ITF and 12 kept as control. During 15 months (April 2010– June 2011) we monitored sand fly species composition and abundance with modified HP light traps inside (domicile) and outside (peridomicile) the studied houses. From 5628 sand flies collected, we were able to identify 5617 of the samples into 24 species, a number of species close to 25±1.6, the estimate from the Chao2 Index. The most abundant species were *Lutzomya trapidoi* (20%), *Lu. gomezi* (20%) and *Lu. triramula* (20%). Cluster analyses showed that most of the 24 houses had high similarity in relative abundance patterns of the six most common species, with only few peripheral houses not following the main cluster pattern. We also found that species richness was decreased to 22 species in the fogged houses, of which only 19 were found in the domiciliary environment. Changes in species richness were especially notorious at the end of the wet season. Our results suggest that species richness can decrease following ITF in domiciliary environments, primarily affecting the less common species.

## Introduction

American Cutaneous Leishmaniasis (ACL) is an increasing public health problem in Panamá, and other neo-tropical countries [1]. This disease affects primarily poor and underserved populations in the region [2], [3]. Besides passive detection and specific treatment of human cases, no measures are currently being undertaken to control the vectors of this parasitic infection. Recently, it has been suggested a change in the epidemiologic pattern of transmission, with the possibility of peridomestic and/or domestic transmission in endemic areas of Panamá [2], moving away from the paradigmatic “sylvatic” transmission [4]. This situation deserves further evaluation, because underappreciated eco-epidemiological changes in transmission could require improved control strategies, with a solid basis on the ecology of the disease, especially the community and population dynamics of sand fly vectors [3].

Phlebotomine sand flies (Diptera: Psychodidae: Phlebotominae) are unique among insect vectors of disease by the co-occurrence of a large number of competent vector species in New World endemic transmission foci of leishmaniasis [4], [5], [6], [7], [8], [9], [10], [11], [12], [13], [14], [15], bartonellosis [16], [17] and vesicular estomatitis [18], [19]. More generally, this group of medically and veterinary important insects is notorious by the co-occurrence of a large number species across the different natural habitats they colonize in the new world [20], [21], [22], [23], [24], [25], [26], [27], such as the tropical agro-forest landscape matrices of Panamá [2], [4], [28], [29], [30], [31], [32], [33].

Sand fly communities have also been documented to undergo major structural changes following landscape transformations or major environmental disturbances. For example, traditional coffee farms are known to host an increased richness of sand fly species, and a relative decreased abundance of major vectors when compared with monoculture coffee farms [34]. Similarly, environmentally degraded environments are known to have a lower richness of sand fly species, and increased abundance of dominant vector species, especially when compared with undisturbed habitats [20], . More generally, it has been suggested that habitat simplification, through vegetation homogenization (i.e., monocultures) or destruction (e.g., deforestation), could underlie the reduction of sand fly species richness and dominant vector species abundance increment [36], a pattern also reported in mosquitoes [37], [38]. For example, resting habitats used by different sand fly species in neotropical forests seem to be very well segregated, i.e., species do not frequently overlap on the use of adult resting sites [29], [39], a pattern in sharp contrast with the overwhelming dominance of few major vector species observed in homogeneous monoculture agricultural landscapes [26], [34].

Interventions with insecticide spraying or fogging are major disturbances that can also potentially modify species composition in a sand fly community. A study conducted 30 years ago in Panamá showed that insecticide fogging in the forest with malathion was able to significantly reduce phlebotomine sand fly density, up to 30% of the abundance, when compared with controls [40]. Nevertheless, that study did not look at changes in species composition following insecticide fogging. Here, we present the results of a 15 month long study where we monitored the phlebotomine faunas of 24 houses, 12 subjected to a couple of domiciliary and peridomiciliary insecticide thermal fogging (ITF) rounds with deltamethrin in the rural village of Trinidad de Las Minas, Capira, Panamá, an hyperendemic focus of American Cutaneous Leishmaniasis (ACL) transmission. This area was selected for an ITF intervention trial because it is an important endemic area for cutaneous leishmaniasis (CL) in Panamá, and because it has never been subjected to any vector control activity. Our aims were to describe the community of vectors in a poorly studied ACL transmission focus of Panamá, and to test whether ITF could change sand fly species composition in the intervened houses.

## Materials and Methods

### Study Area

**Figure 1:**
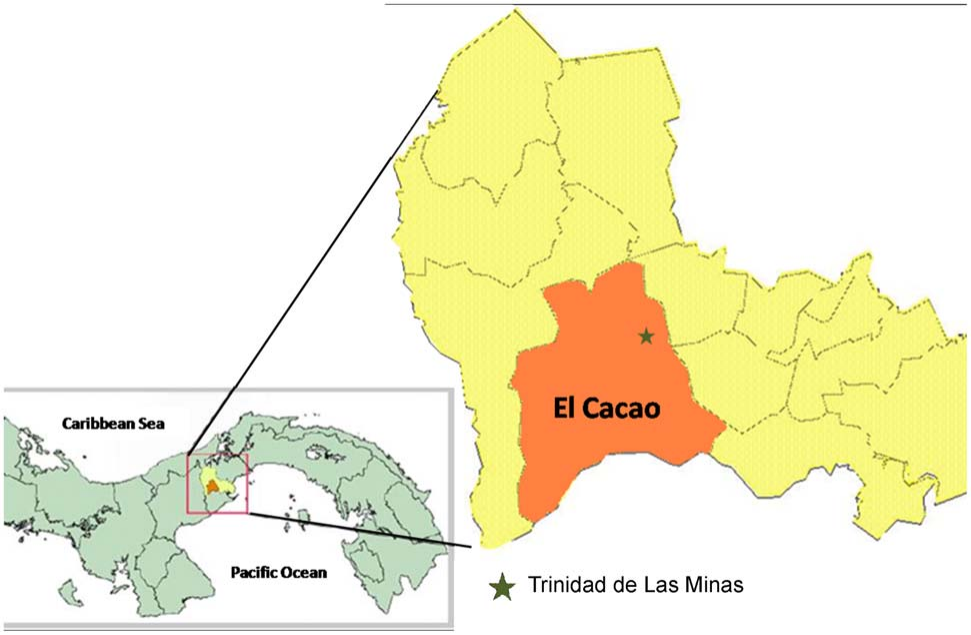
Map of Panamá, showing the location of Trinidad de Las Minas, Capira, El Cacao County, Capira District, Western Panamá province.

The study was conducted between April 2010– June 2011 in the rural village of Trinidad de Las Minas, (8°46′32′′N and 79°59′45′′W), located in the western region of Panamá Province, Capira District, Panamá (Fig. 1). This village is located 230 meters above sea level, with an annual mean temperature of 26.0°C and monthly rainfall ranging 28–570 mm^3^. Climate is characterized by a marked seasonality, with a dry season from mid December to March and a rainy season during all other months. This area is ecologically classified as lowland tropical moist forest. During recent years regional native vegetation has been destroyed mainly for agricultural development, and the forest has become transitional, with some deciduous xerophile species.

We selected 24 houses for our study (out of 128 houses in the village). Twelve houses were subjected to indoor and outdoor insecticide thermal fogging, while the remaining 12 houses were kept as control (no fogging). The number of houses evaluated in this study was limited by the resources available for this study, especially the availability of light traps. Studied houses were located in a village area with similar eco-epidemiological conditions, where the presence of sand flies had been previously confirmed and where residents provided consent to participate in the study. Selection of houses for the fogging was constrained by participant consent, which prevented a fully randomized or matched assignation of houses into the control or fogged groups. Nevertheless, we consider our study design was sound to test for differences in sand fly species composition given the low dispersal ability of sand flies, which, in general, is below 50 m from the release site [41],[42].

#### Ethical Clearance

This study was approved by the National Review Board, Comité Nacional de Bioética de la Investigación, Instituto Conmemorativo Gorgas de Estudios de la Salud, Panamá City, Panamá (561/CNBI/ICGES/06).

### Sand Fly Collection And Identification

**Figure 2:**
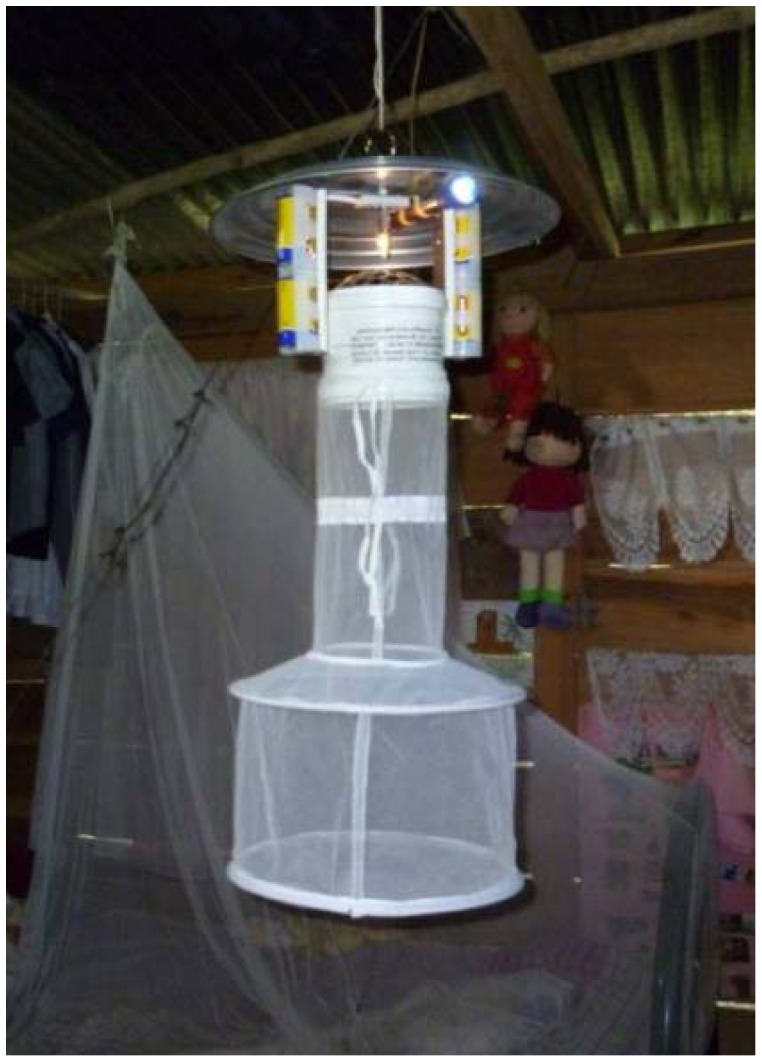
LED light modified HP trap.

Sand flies were collected using HP light-traps (See Fig. 2). Each trap was slightly modified by attaching an additional small LED light to increase sand fly attraction. Entomological samplings were carried out monthly from April 2010 to June 2011, except for the months of August and November 2010 and January 2011, when access to this remote village was impossible because of logistical and operational constraints, which prevented the sampling of houses before the 2^nd^ ITF. Thus, a total of 12 sampling surveys were conducted during the study. For each monthly collection, one trap was placed for one night in the main bedroom of every household (indoor). This trap was suspended from the ceiling at about 2 m from the ground floor. Another trap was placed at the same height, above vegetation, within 50 meters of the house (i.e., peridomicile). Traps were setup for 12 hours, from 6∶00 pm to 6∶00 am, in the same position (indoor and peridomicile) during each sampling session. In total there were 24 trap-nights of sampling per house, 12 inside each house, 12 in the peridomicile.

Trapped sand flies were removed from the traps, stored at −20°C to kill the samples and preserved in 70% ethanol for identification. For each trap we summarized the abundance, sex and species of sand flies using the taxonomic guide of Young and Duncan [43], with male genitalia and female spermathecae as main diagnostic taxonomic characters.

### Insecticide Thermal Fogging

**Figure 3:**
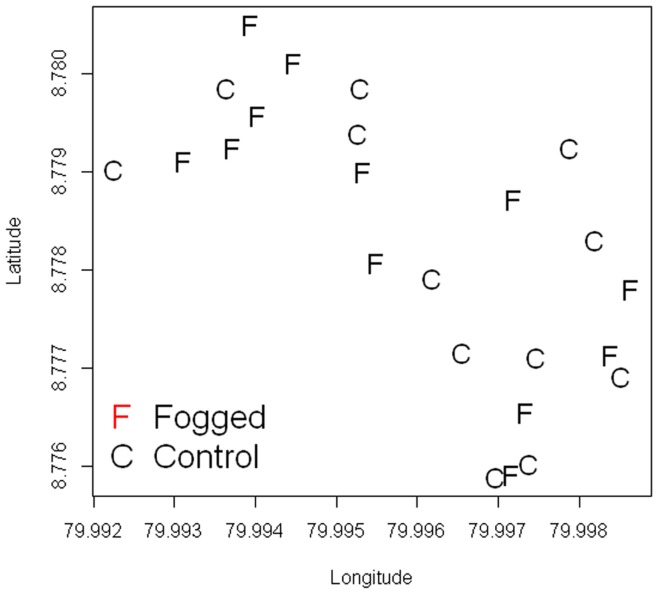
Control and insecticide fogged houses at Trinidad de Las Minas, Capira. In the y and x axis 0.001 degree of latitude/longitude are approximately 110 m.

The vector control intervention for evaluation consisted of two rounds of indoor/outdoor insecticide thermal fogging (ITF) using deltamethrin (K-Othrine® 2.7 UBV, Bayer, Guatemala) in the intervened (fogged group). Insecticide selection and application was performed by trained personnel of the Vector Control Department from the Ministry of Health. Following National guidelines, deltamethrin was diluted in diesel to a final concentration of 0.7 g/L. The insecticide applications were conducted on July 18, 2010 and January 23, 2011. The insecticide was applied with a hand-held thermal fogger (Golden EagleTM, Model # 2610, Curtis Dyna-Fog Ltd, Westfield, IN, USA) to interior and exterior housing walls, targeting cracks and crevices. A similar fogging was performed in the 15 m around the houses (peridomicile). Location of fogged and control houses can be seen in Fig. 3.

### Statistical Analysis

#### Species Number Estimation

To estimate the number of species and to evaluate how comprehensive was our sampling of the Sand Fly fauna with the HP light traps, we used the abundance data from each trap, using a species accumulation curve with inference based on the rarefaction method [44]. The species accumulation curve, SAC, estimates the expected species richness and its standard deviation by sampling individuals based on their abundance per sampling effort unit (i.e., rarefaction). We employed the SAC for (i) all the traps, (ii) the domiciliary and peridomiciliary traps and (iii) the domiciliary and peridomiciliary traps of the fogged and control houses. We also estimated the cumulative Chao2 index for all the traps. With the Chao2 index species richness estimation is based on the cumulative incidence matrix of species across the sampled sites, i.e., a matrix that summarizes the patterns of absence/presence of each species across the sampled locations, thus being independent of species abundance [45].

#### Patterns Of Species Clustering In The Community Of Sand Fly Species

We performed an analysis to consider both the possibility of spatial clustering of sand fly species abundance patterns and also to quantify similarities in the sand fly fauna.

#### Spatial Analysis

To test whether community species abundance patterns were homogeneous across the houses in our study village, we performed a multinomial scan spatial clustering analysis [46] for the total abundance, i.e., the summation of all our samples, for the six most common species and a category composed by all other species. Briefly, the multinomial scan spatial clustering test examines the relative abundance of counts from several categories of data, i.e., a multinomial distribution [46]. For the six most abundant vector species and the category for the remainder of the species, the test compares the observed values versus the expectation of a homogenous distribution in a circumference of a given radius. The procedure is repeated across the landscape containing the observations, testing several radii below a maximum, and the circumference for which the likelihood is maximized is the most likely cluster [46].

#### Species Similarity Analysis

We also studied the similarity of species composition across the different houses. For this purpose we computed the Sørensen similarity index, an index between 0 and 1 where high values imply a high similarity in species composition; i.e., 1 means that all species are shared between two sampling locations, i.e., houses in this study, and 0 the total lack of similar species between two sites [47]. To ease the visualization of our results we performed a cluster analysis. Briefly, cluster analysis is a multivariate of analysis in which elements of a dataset are arranged in groups or subsets based on their characteristics. In our analysis, the characteristic employed for cluster construction was the Sørensen index computed between the different pairs of households (i.e, the “elements” analyzed in the cluster analysis). We employed an agglomerative hierarchical cluster technique in which elements (i.e., households) were joined with the most similar elements (i.e., other households) during iterative steps of a joining algorithm. For the clustering, a complete linkage algorithm was used in order to find very similar clusters [48]. We also employed the values of the Sørensen index to test any potential effects of distance on the species composition similarity as function of the distance between the houses.

#### Temporal Patterns Of Species Richness And Diversity Evenness

We used the monthly data from each site to estimate the monthly species richness, i.e., the number of species, and the Shannon index for the fogged and control houses. The Shannon index is a diversity evenness index, i.e., a measure of differences in the relative abundance of species in a community, where low values indicate that a community is dominated by relatively fewer species than communities with higher values, where a more equitative abundance of species is observed [47].

#### Statistical Software

All the analyses, with the exception of the multinomial SCAN, were performed with the package vegan for the statistical software R version 2.0.14. The multinomial SCAN analysis was performed with the statistical software SaTScan.

## Results

During the 15 months of our study we collected 5628 sand flies, from which we were able to identify 5617 of the samples into 24 species, 23 belonging to the genus *Lutzomyia* and 1 *Brumptomyia* (Table 1). Table 1 shows the detailed account of species and their abundance. Sand flies were more frequently collected outside (peridomicile) than inside (domicile) the studied houses (58% vs 42%).The most abundant antropophilic species were *Lutzomyia trapidoi* (20%) *Lu. gomezi* (20%) and *Lu. panamensis* (17%). The most frequent zoophilic species were *Lu. triramula* (20%) and *Lu. dysponeta* (8.7%). Table 1 also shows that total abundance of sand flies was reduced by about 40% inside the intervened houses (fogged), both inside the houses (i.e., domicile; control: 1484, fogged: 881) and by 50% in the peridomiciliary environments (control: 2157, fogged: 1106).

**Table 1:** Total sand fly species abundance in control and fogged houses.

Species	Vector status	Control		Fogged		Total
		Domicile	Peridomicile	Domicile	Peridomicile	%
**Anthropophilic**						
*Lutzomyia trapidoi* (Fairchild & Hertig)	Y	562	158	228	203	20.45
*Lu. gomezi* (Nitzulescu)	Y	448	238	291	169	20.36
*Lu. panamensis* (Shannon)	Y	99	470	88	310	17.18
*Lu. olmeca* (Vargas & Díaz Nájera)	Y	33	55	14	60	2.88
*Lu. ylephiletor* (Fairchild & Hertig)	Y	2	8	0	5	0.27
*Lu. shannoni* (Dyar)	Y	10	0	5	3	0.32
*Lu. sanguinaria* (Fairchild & Hertig)	U	6	1	2	1	0.18
*Lu. ovallesi* (Ortiz)	Y	97	15	47	7	2.95
**Zoophilic**						
*Lu. triramula* (Fairchild & Hertig)	U	71	902	25	152	20.43
*Lu. dysponeta* (Fairchild & Hertig)	U	67	193	126	104	8.71
*Lu. camposi* (Rodriguez)	U	22	55	16	29	2.17
*Lu. vesicifera* (Fairchild & Hertig)	U	15	16	7	21	1.05
*Lu. sordelli* (Mangabeira Fo)	U	1	4	2	8	0.27
*Lu. vespertilionis* (Fairchild & Hertig)	U	1	7	0	4	0.21
*Lu. carpenteri* (Fairchild & Hertig)	U	5	22	6	14	0.84
*Brumptomyia hamata* (Fairchild & Hertig)	U	2	0	2	1	0.09
*Lu. serrana* (Damasceno & Arouck)	U	16	6	9	3	0.60
*Lu. furcata* (Mangabeira Fo)	U	1	0	0	0	0.02
*Lu. gorbitzi* (Blancas)	U	17	0	7	1	0.44
*Lu. aclydifera* (Fairchild & Hertig)	U	0	1	0	2	0.05
*Lu. carrerai thula* Young	U	1	4	0	4	0.16
*Lu. punctigeniculata* (Floch & Abonnec)	U	4	1	2	1	0.14
*Lu. cruciata* (Coquillet)	U	1	0	0	0	0.02
*Lu. trinidadensis* (Newstead)	U	1	0	0	0	0.02
Unidentified	–	2	1	4	4	0.20
**Total**		1484	2157	881	1106	

The bottom row shows the total number of individuals sampled in each category. A total of 24 houses were monitored (12 as control and 12 for the insecticide thermal fogging intervention) and each house underwent a total sampling effort of 24 trap nights (12 Domicile and 12 Peridomicile). Vector Status indicates proven *Leishmania* parasitic development in field and/or laboratory conditions (Y) and unknown (U) according to Christensen et al [4], Lawyer et al [60] and Feliciangeli et al [61].

doi:10.1371/journal.pone.0053289.t001

**Figure 4:**
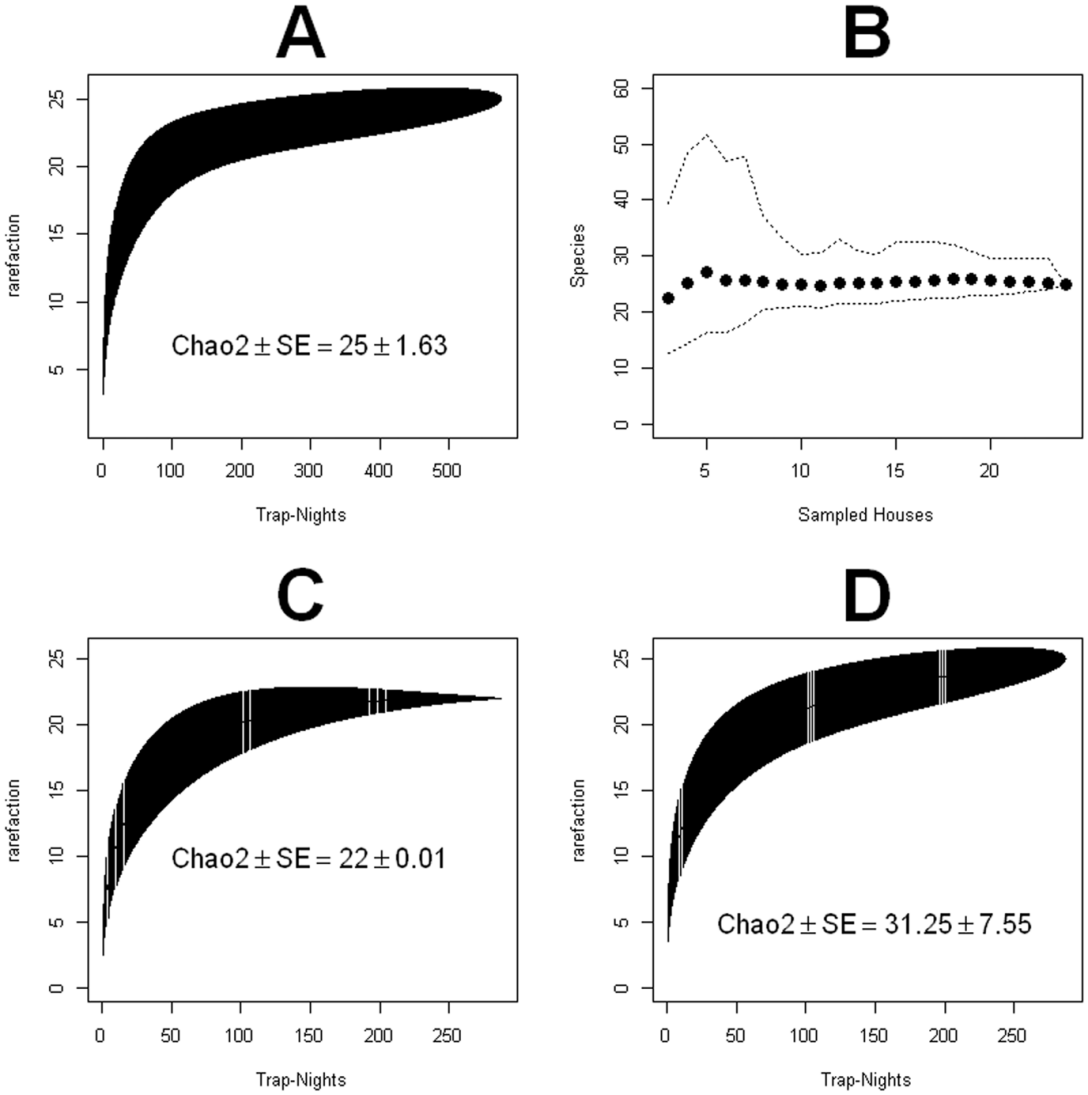
Sand fly diversity sampling (A) Species accumulation curve for all the traps (peridomicilary and domiciliary, control and fogged) (B) Chao2 index on the cumulative incidence of species in the traps monitored during the study period, dotted lines are for the 95% confidence intervals and dots represent the estimates (C) Species accumulation curve for all the traps (domiciliary and peridomiciliary) of the fogged houses (D) Species accumulation curve for all the traps (domiciliary and peridomiciliary) of the control houses. (A), (C) and (D) present results for the rarefaction based species accumulation curve as function of trap nights.

**Figure 5:**
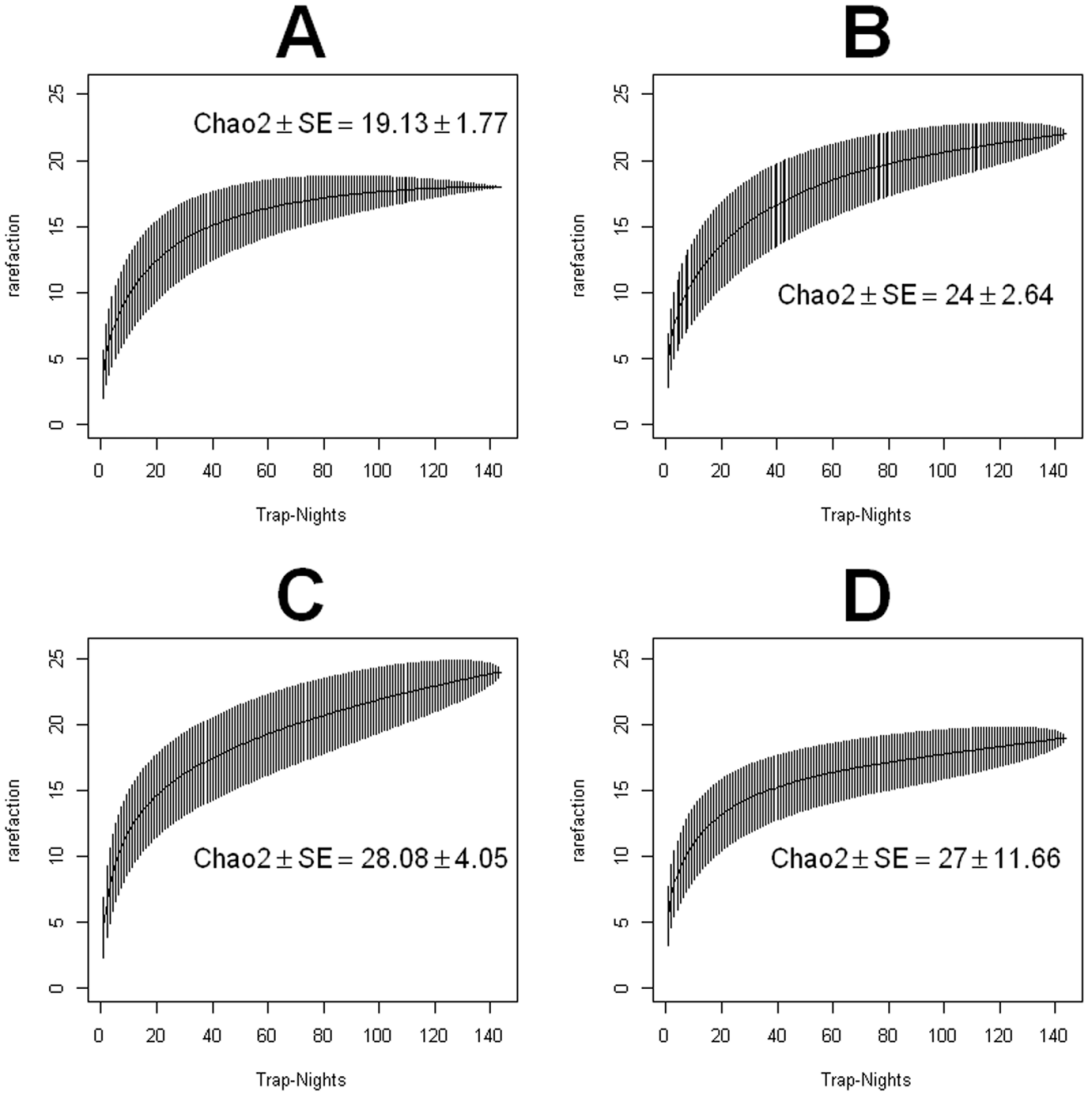
Domiciliary and Peridomiciliary phlebotomine sand fly species richness in control and fogged houses. Species accumulation curve for (A) Fogged Domicile (B) Fogged Peridomicile (C) Control Domicile, (D) Control Peridomicile.).

Species accumulation curves (SAC) showed that our sampling of sand fly species diversity was very comprehensive. The flat slope of the SAC after 400 trap nights (Fig. 4A), the plateau of 25 species in the SAC, the Chao2 estimate of 25 species, and the convergence of the cumulative Chao2 index to 25 species, when all houses were considered in the analysis (Fig. 4B), were all values extremely close to the 24 species that we recorded, which could potentially be 25 assuming some of the unidentifiable individuals belonged to a different species. A similar pattern was observed for the richness of the fogged houses sand fly fauna, where the SAC converged with the observed 22 species (Fig. 4C), as well as with the Chao2 index estimate. Fig. 4D shows the convergence of the SAC from the control houses with the 24 observed species, an estimate also contained within the estimates of the Chao2 index. When we further split the traps by control and fogged and by domicile and peridomicile (Fig. 5), we found a perfect agreement between the 19 recorded species and the predictions by the Chao2 and the SAC for the domiciliary species of the fogged houses (Fig. 5A). The 23 recorded peridomiciliary species in the fogged houses were also within the boundaries of the predictions by the SAC and Chao2 index (Fig. 5B), so were the 24 species recorded in the domicile of the control houses (Fig. 5C) and the 19 species observed in the peridomicile of the control houses (Fig. 5D).

**Figure 6:**
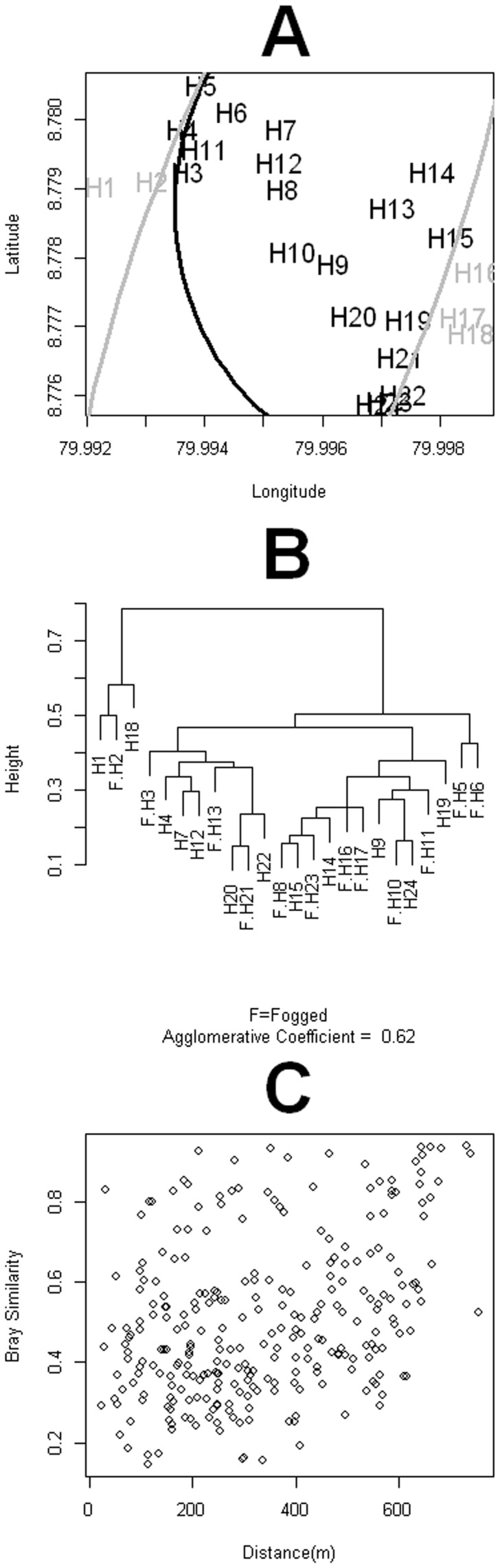
Study setting and similarities in sand fly species composition (A) Multinomial clusters, the black and grey solid lines are, respectively, the multinomial cluster boundaries for the 6 most abundant species in the domiciles and peridomiciles. Black symbols represent houses inside the two clusters and grey symbols represent houses outside at least one cluster (B) Agglomerative clusters of the Sørensen index for species similarity in the studied houses (C) Sørensen index of species similarity as function of between house distance.

The multinomial scan spatial statistics showed the six most abundant species had similar proportions across most of the houses (Table 2, Fig. 6A). Nevertheless, H1 and H2 were consistently different from all of the other houses, both in the domiciliary and peri-domiciliary faunas (Fig. 6A, Table 2). Houses H16, H17, H18 presented peridomiciliary faunas that were significantly different from other houses (Fig. 6A, Table 2). The cluster of species composition similarity (Fig. 6B) also showed that H1, H2 and H18 had faunas different from those of the other houses. However, most of the houses presented a very similar species composition, independently of whether they were fogged or not, even if comparisons were restricted to the records observed after the beginning of the intervention (not shown). The species composition similarity between houses was independent of their geographic distance (Fig. 6C).

**Table 2:** Multinomial Scan Statistics.

Environment	Houses	Sand Fly speciesabundance	Observed/Expected	Log-Likelihood	P
Domicile	H13, H14, H15, H9, H19, H20,H16, H10, H8, H17, H12, H21, H7,H18, H22, H23, H24, H6, H11, H3, H5, H4	T = 2052, A = 194,B = 284, C = 150, D = 29,E = 56, F = 190	A = 0.56,B = 0.87, C = 1.82,D = 1.40, E = 1.33, F = 2.24	269.96	0.001
Peridomicile	H10, H8, H9, H12, H7, H20, H13,H6, H11, H14, H3, H19, H24, H21,H23, H5, H4, H15, H22	T = 3014, A = 260,B = 282, C = 403, D = 69,E = 143, F = 200	A = 1.60, B = 1.54, C = 1.15,D = 1.33, E = 0.30, F = 1.50	385.46	0.001

Here we show the main clusters of the six most abundant species in the domicile and peridomicile (Environment). Houses indicate the houses included in each spatial cluster, sand fly abundance are the observed counts and observed/expected the ratio between the sand fly counts and the expectation from the null model with a spatially homogeneous multinomial distribution. T = total, A = *Lutzomyia trapidoi*, B = *Lu. gomezi,* C* = Lu. panamensis,* D = *Lu. olmeca,* E* = Lu. triramula,* F* = Lu. dysponeta*. For the analysis we assumed the maximum cluster size to be one covering 50% of the sampled sand flies and we allowed the clusters to have ellipsoidal shapes.

doi:10.1371/journal.pone.0053289.t002

**Figure 7:**
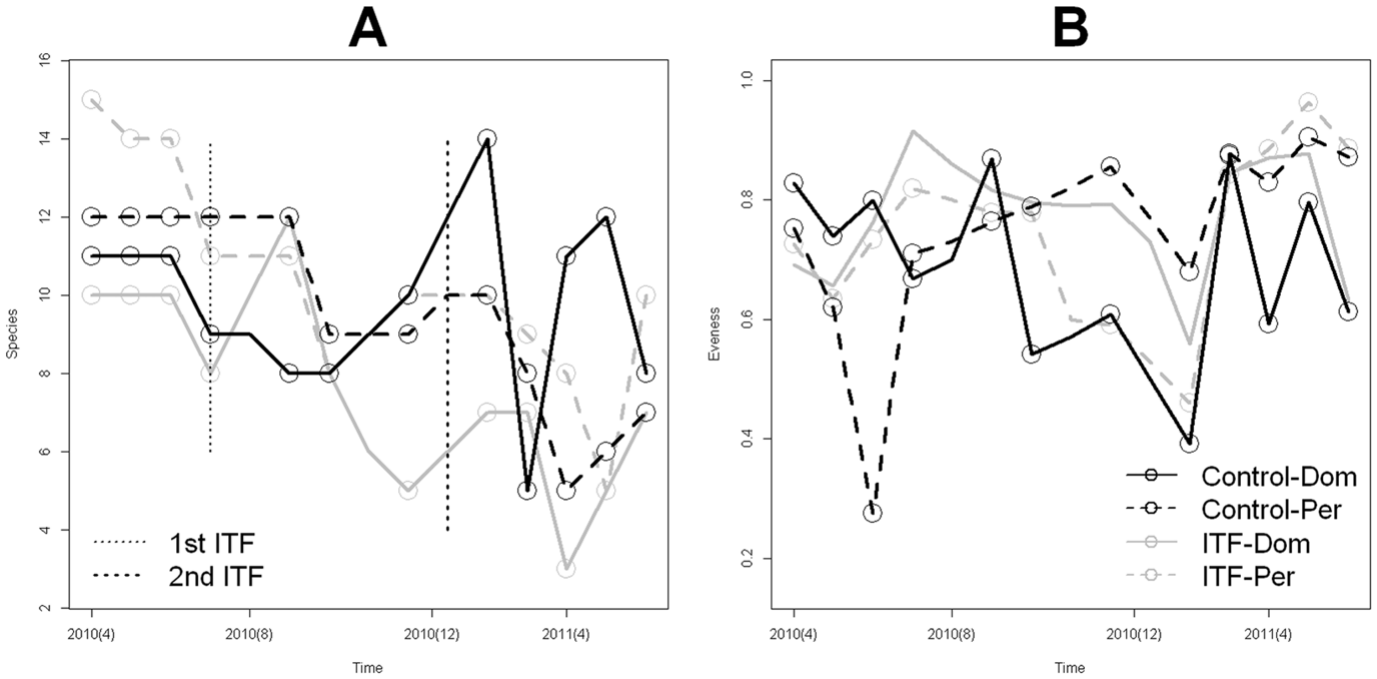
Temporal dynamics of phlebotomine sand fly species (A) Richness (B) Evenness in the domiciliary (Dom) and peridomiciliary (Per) environments of the control and fogged houses. In (A) dotted vertical lines indicate the timing of the foggings with deltamethrin.

The temporal dynamics of species richness (Fig. 7A) showed that following the foggings there was a decrease in species richness in the domiciliary and peri-domiciliary environments of the fogged houses, which was more transient, i.e., returned to comparable levels with the control more quickly, in the peridomicile. However, there were no concomitant changes in the evenness of the communities following the interventions (Fig. 7B); i.e., relative abundance and dominance of vector species was similar before and after the interventions, even though total sand fly abundance and richness decreased.

## Discussion

Results presented in Table 1 in conjunction with Fig. 4 and Fig. 5 support that our sampling of species richness was comprehensive. In our studied area, we found 24 of the 74 native species of phlebotomine sand flies reported for Panamá [4],[33],[43],[49] and the slope of the species accumulation curve, for all the traps (Fig. 4A), flattened as expected when an exhaustive sampling is done [44]. A similar curve slope flattening behavior was observed for the cumulative Chao2 index [45],[50], a result that further reinforces our confidence in the quality of our species richness sampling with HP light traps. In this context we want to emphasize that previous studies in sand flies have shown that light traps are very reliable to sample species richness of sand flies when compared with sticky traps [51], aspiration of resting sites [29] and aspiration from Shannon traps [52]. Nevertheless, abundance can be variable depending on the kind of trap used [53],[54],[55], yet HP traps have been reported as very effective to efficiently sample large number of new world sand flies [56], which further makes us confident in the quality of the data used for our richness estimates.

The most abundant species in our samples were *Lutzomyia triramula* (20%), *Lu. gomezi* (20%), *Lu. trapidoi* (20%) and *Lu. panamensis* (17%), the last three species are known to be highly competent vectors of *Leishmania* spp parasites in Panamá [2],[4],[49]. Other recognized vectors of *Leishmania* spp parasites to humans we collected were *Lu. olmeca* (3%), *Lu. ovallesi* (3%), *Lu. shannoni* (0.3%) and *Lu. ylephiletor* (0.3%) [4]. Species composition across all houses was very similar, as demonstrated both by patterns of spatial clustering in the six most common species (Table 1, Fig. 6B) and in the clustering patterns of Sørensen similarity indices (Fig. 6C). This is an interesting pattern, which is along the lines of patterns described in Panamá [32],[57] and Venezuela [8] where sand fly species co-occur more often than what would be expected by random. We also found *Lu. panamensis* and *Lu. gomezi* frequently co-occurring in our samples, a pattern previously described for these two species of high vectorial capacity for the transmission of *Leishmania (Viannia) panamensis* [32], the main parasite causing cutaneous leishmaniasis in Panamá [2],[4].

Our data shows that houses subjected to ITF had 40% the abundance of domiciliary and peridomiciliary sand flies observed in the control houses, a reduction similar to the average reduction recorded in a previous study with fogging in a tropical forest environment of Panamá, which employed malathion as insecticide [40]. Thus, our results can be considered on the side of successful ITF interventions, especially in light of several studies reporting non-significant reductions in sand fly abundance following insecticide based trial interventions worldwide [58]. Our focus here was to describe the sand fly community species composition at a rural village in western Panamá, and to test whether ITF was able to induce changes in the species composition of a community of sand flies not exposed to any previous ITF intervention. In a separate study we will present an in depth analysis of the impact of ITF on dominant phlebotomine sand fly vector species abundance.

Our species richness analysis indicates that reduction on sand fly abundance observed in intervened houses led to a statistically significant reduction (mean ± S.E. do not overlap) in the number of sand fly species collected on the domiciliary environment (Table 1) of the intervened houses (Fig. 5A), when compared with the richness of the control houses (Fig. 5C). An inspection of Table 1 suggests that differences in richness where due to the absence of rare species inside the sprayed houses. By contrast, in peri-domiciliary environments there were not significant differences in species richness (Figs. 5B, 5D). The latter pattern probably underlies the high degree of species similarity across houses (Fig. 6A, 6B). If we look at the temporal patterns of species richness we found that following the ITF rounds species richness decreased in both the domicile and peridomicile (Fig. 7A). Nevertheless, diversity, as measured by the Shannon index, was very stable through our study period (Fig. 7B), indicating that ITF does not seem to affect the proportional patterns of species abundance, a result congruent with the clustering of most of the houses both spatially and in species similarity we described earlier.

In conclusion, our results show that ITF in ACL endemic areas are able to diminish the abundance of phlebotomine sand flies and species richness, especially inside intervened houses (domicilary environments). Although we did not find a significant difference in species richness outside the houses (peridomiciliary environments) we consider that future trials may be able to show differences in species richness, especially if more sophisticated trial designs [59], which were beyond the available resources for our study, are implemented.

## References

[1] Alvar J, Vélez ID, Bern C, Herrero M, Desjeux P, et al (2012) Leishmaniasis Worldwide and Global Estimates of Its Incidence. PLoS ONE 7: e35671. 22693548 10.1371/journal.pone.0035671 PMC3365071

[2] Miranda A, Carrasco R, Paz H, Pascale JM, Samudio F, et al (2009) Molecular Epidemiology of American Tegumentary Leishmaniasis in Panama. Am J Trop Med Hyg 81: 565–571. 19815867 10.4269/ajtmh.2009.08-0265

[3] Chaves LF, Cohen JM, Pascual M, Wilson ML (2008) Social Exclusion Modifies Climate and Deforestation Impacts on a Vector-Borne Disease. PLoS Negl Trop Dis 2: e176. 18265876 10.1371/journal.pntd.0000176 PMC2238711

[4] Christensen HA, Fairchild GB, Herrer A, Johnson CM, Young DG, et al (1983) The ecology of cutaneous leishmaniasis in the republic of Panama. J Med Entomol 20: 463–484. 6358496 10.1093/jmedent/20.5.463

[5] Feliciangeli MD (1987) Ecology of sandflies (Diptera: Psychodidae) in a restricted focus of cutaneous leishmaniasis en Northern Venezuela: I. Description of the study area, catching methods and species composition. Mem Inst Oswaldo Cruz 82: 119–124. 3507559 10.1590/s0074-02761987000100019

[6] Feliciangeli MD (1987) Ecology of sandflies (Diptera: Psychodidae) in a restricted focus of cutaneous leishmaniasis in Northern Venezuela: II. Species composition in relation to habitat, catching method and hour of catching. Mem Inst Oswaldo Cruz 82: 125–131. 10.1590/s0074-02761987000100020 3507560

[7] Alexander B, Usma MC, Cadena H, Quesada BL, Solarte Y, et al (1995) Phlebotomine sandflies associated with a focus of cutaneous leishmaniasis in Valle del Cauca, Colombia. Med Vet Entomol 9: 273–278. 7548944 10.1111/j.1365-2915.1995.tb00133.x

[8] Chaves LF, Añez N (2004) Species co-occurrence and feeding behavior in sand fly transmission of American cutaneous leishmaniasis in western Venezuela. Acta Trop 92: 219–224. 15533290 10.1016/j.actatropica.2004.08.001

[9] Feliciangeli MD, Rodriguez N, Bravo A, Arias F, Guzman B (1994) Vectors of cutaneous leishmaniasis in north-central Venezuela. Med Vet Entomol 8: 317–324. 7841486 10.1111/j.1365-2915.1994.tb00095.x

[10] Salomon OD, Wilson ML, Munstermann LE, Travi BL (2004) Spatial and Temporal Patterns of Phlebotomine Sand Flies (Diptera: Psychodidae) in a Cutaneous Leishmaniasis Focus in Northern Argentina. J Med Entomol 41: 33–39. 14989343 10.1603/0022-2585-41.1.33

[11] Salomon OD, Rosa JR, Stein M, Quintana MG, Fernandez MS, et al (2008) Phlebotominae (Diptera: Psycodidae) fauna in the Chaco region and Cutaneous Leishmaniasis transmission patterns in Argentina. Mem Inst Oswaldo Cruz 103: 578–584. 18949328 10.1590/s0074-02762008000600011

[12] Salomon OD, Quintana MG, Zaidenberg M (2008) Urban distribution of Phlebotominae in a cutaneous leishmaniasis focus, Argentina. Mem Inst Oswaldo Cruz 103: 282–287. 18545855 10.1590/s0074-02762008005000016

[13] Carvalho GM, Gontijo CM, Falcao AL, Andrade Filho JD (2010) Study of Phlebotomine Sand Flies (Diptera: Psychodidae) Collected in a *Leishmania*-Endemic Area of the Metropolitan Region of Belo Horizonte, Brazil. J Med Entomol 47: 972–976. 21175043 10.1603/me09127

[14] Margonari C, Soares RP, Andrade-Filho JD, Xavier DC, Saraiva L, et al (2010) Phlebotomine Sand Flies (Diptera: Psychodidae) and *Leishmania* Infection in Gafanhoto Park, Divinopolis, Brazil. J Med Entomol 47: 1212–1219. 21175074 10.1603/me09248

[15] Jimenez AE, Rojas JC, Vargas F, Herrero MV (2000) Temporal and Spatial Variation of Phlebotomine (Diptera: Psychodidae) Community Diversity in a Cutaneous Leishmaniasis Endemic Area of Costa Rica. J Med Entomol 37: 216–221. 10730490 10.1603/0022-2585-37.2.216

[16] Herrer A, Christensen HA (1975) Implication of Phlebotomus Sand Flies as Vectors of Bartonellosis and Leishmaniasis as Early as 1764. Science 190: 154–155. 1101379 10.1126/science.1101379

[17] Alexander B (1995) A review of bartonellosis in Ecuador and Colombia. Am J Trop Med Hyg 52: 354–359. 7741177 10.4269/ajtmh.1995.52.354

[18] Herrero MV, Jimenez AE, Rodriguez LL, Pereira R (1994) Phlebotomines (Diptera: Psychodidae) Collected at a Costa Rican Dairy Farm in a Vesicular Stomatitis Endemic Area. J Med Entomol 31: 912–914. 7815407 10.1093/jmedent/31.6.912

[19] Schmidtmann ET, Craig ME, English LM, Herrero MV (2002) Sampling for Sand Flies (Diptera: Psychodidae) Among Prairie Dog Colonies on Ranches with Histories of Vesicular Stomatitis in New Mexico and Colorado. J Med Entomol 39: 680–684. 12144304 10.1603/0022-2585-39.4.680

[20] Fernández MS, Lestani EA, Cavia R, Salomón OD (2012) Phlebotominae fauna in a recent deforested area with American Tegumentary Leishmaniasis transmission (Puerto Iguazú, Misiones, Argentina): Seasonal distribution in domestic and peridomestic environments. Acta Trop 122: 16–23. 22155061 10.1016/j.actatropica.2011.11.006

[21] Pinto IS, dos Santos CB, Ferreira AL, Falqueto A (2010) Richness and diversity of sand flies (Diptera, Psychodidae) in an Atlantic rainforest reserve in southeastern Brazil. J Vector Ecol 35: 325–332. 21175939 10.1111/j.1948-7134.2010.00090.x

[22] De Lima Carvalho GM, De Vasconcelos FB, Da Silva DG, Botelho HA, Andrade Filho JD (2011) Diversity of Phlebotomine Sand Flies (Diptera: Psychodidae) in Ibitipoca State Park, Minas Gerais, Brazil. J Med Entomol 48: 764–769. 21845934 10.1603/me10258

[23] Ximenes MdF, De Melo F, Castellon EG, de Souza MdF, Freitas RA, et al (2000) Distribution of Phlebotomine Sand Flies (Diptera: Psychodidae) in the State of Rio Grande do Norte, Brazil. J Med Entomol 37: 162–169. 15218921 10.1603/0022-2585-37.1.162

[24] Salomon OD, Rossi GC, Cousino B, Spinelli GR, de Arias AR, et al (2003) Phlebotominae sand flies in Paraguay. Abundance distribution in the southeastern region. Mem Inst Oswaldo Cruz 98: 185–190. 12764432 10.1590/s0074-02762003000200004

[25] Ferro C, Morrison AC, Torres M, Pardo R, Wilson ML, et al (1995) Species Composition and Relative Abundance of Sand Flies of the Genus *Lutzomyia* (Diptera: Psychodidae) at an Endemic Focus of Visceral Leishmaniasis in Colombia. J Med Entomol 32: 527–537. 7650716 10.1093/jmedent/32.4.527

[26] Quintana MG, Salomon OD, De Grosso M, Lizarralde S (2010) Distribution of Phlebotomine Sand Flies (Diptera: Psychodidae) in a Primary Forest-Crop Interface, Salta, Argentina. J Med Entomol 47: 1003–1010. 21175047 10.1603/me09072

[27] Rotureau B, Gaborit P, Issaly J, Carinci R, Fouque F, et al (2006) Diversity and ecology of Sand Flies (Diptera: Psychodidae: Phlebotominae) in coastal French Guiana. Am J Trop Med Hyg 75: 62–69. 16837710

[28] Azpurua J, De La Cruz D, Valderama A, Windsor D (2010) *Lutzomyia* Sand Fly Diversity and Rates of Infection by *Wolbachia* and an Exotic *Leishmania* Species on Barro Colorado Island, Panama. PLoS Negl Trop Dis 4: e627. 20231892 10.1371/journal.pntd.0000627 PMC2834748

[29] Chaniotis BN, Tesh RB, Correa MA, Johnson KM (1972) Diurnal resting sites of phlebotomine sandflies in a Panamanian tropical forest. J Med Entomol 9: 91–98. 5019215 10.1093/jmedent/9.1.91

[30] Rutledge LC, Ellenwood DA (1975) Production of Phlebotomine Sandflies on the Open Forest Floor in Panama: The Species Complement. Environ Entomol 4: 71–77.

[31] Rutledge LC, Walton BC, Ellenwood DA, Correa MA (1976) A Transect Study of Sand Fly Populations in Panama (Diptera, Psychodidae). Environ Entomol 5: 1149–1154.

[32] Rutledge LG, Ellenwood DA, Johnston L (1975) An analysis of Sand Fly Light trap collections in the Panama canal zone (Diptera: Psychodidae). J Med Entomol 12: 179–183. 1159739 10.1093/jmedent/12.2.179

[33] Fairchild GB, Hertig M (1959) Geographic distribution of the Phlebotomus Sandflies of Central America (Diptera:Psychodidae). Ann Entomol Soc Am 52: 121–124.

[34] Alexander B, Agudelo LA, Navarro F, Ruiz F, Molina J, et al (2001) Phlebotomine sandflies and leishmaniasis risks in Colombian coffee plantations under two systems of cultivation. Med Vet Entomol 15: 364–373. 11776455 10.1046/j.0269-283x.2001.00322.x

[35] Travi BL, Adler GH, Lozano M, Cadena H, Montoya-Lerma J (2002) Impact of Habitat Degradation on Phlebotominae (Diptera: Psychodidae) of Tropical Dry Forests in Northern Colombia. J Med Entomol 39: 451–456. 12061439 10.1603/0022-2585-39.3.451

[36] Chaves LF (2011) Sand fly species co-occurrence at the local scale: Differences between agricultural and forested areas. Bol Malariol Salud Amb 51: 35–39.

[37] Chaves LF, Hamer GL, Walker ED, Brown WM, Ruiz MO, et al (2011) Climatic variability and landscape heterogeneity impact urban mosquito diversity and vector abundance and infection. Ecosphere 2: art70.

[38] Gleiser RM, Zalazar LP (2010) Distribution of mosquitoes in relation to urban landscape characteristics. Bull Ent Res100: 153–158. 10.1017/S0007485309006919 19413916

[39] Valderrama A, Tavares MG, Andrade Filho JD (2011) Anthropogenic influence on the distribution, abundance and diversity of sandfly species (Diptera: Phlebotominae: Psychodidae), vectors of cutaneous leishmaniasis in Panama. Mem Inst Oswaldo Cruz 106: 1024–1031. 22241128 10.1590/s0074-02762011000800021

[40] Chaniotis BN, Parsons RE, Harlan HJ, Correa MA (1982) A pilot study to control phlebotomine Sand Flies (Diptera: Psychodidae) in a Neotropical Rain Forest J Med Entomol. 19: 1–5. 10.1093/jmedent/19.1.1 7120291

[41] Alexander JB (1987) Dispersal of Phlebotomine Sand Flies (Diptera: Psychodidae) in a Colombian Coffee Plantation. J Med Entomol 24: 552–558. 3669027 10.1093/jmedent/24.5.552

[42] Morrison AC, Ferro C, Morales A, Tesh RB, Wilson ML (1993) Dispersal of the Sand Fly Lutzomyia longipalpis (Diptera: Psychodidae) at an Endemic Focus of Visceral Leishmaniasis in Colombia. J Med Entomol 30: 427–435. 8459421 10.1093/jmedent/30.2.427

[43] Young DG, Duncan MA (1994) Guide to the identification and geographic distribution of *Lutzomyia* sand flies in Mexico, the West Indies, Central and South America (Diptera: Psychodidae). Gainesville, FL: Associated Publishers. 881 p.

[44] Colwell RK, Coddington JA (1994) Estimating Terrestrial Biodiversity through Extrapolation. Phil Trans Roy Soc London Ser B-Biol Sci 345: 101–118. 7972351 10.1098/rstb.1994.0091

[45] Chao A, Chazdon RL, Colwell RK, Shen TJ (2005) A new statistical approach for assessing similarity of species composition with incidence and abundance data. Ecol Lett 8: 148–159.

[46] Jung I, Kulldorff M, Richard OJ (2010) A spatial scan statistic for multinomial data. Stat Med 29: 1910–1918. 20680984 10.1002/sim.3951 PMC4147837

[47] Krebs CJ (1998) Ecological Methodology: Benjamin Cummings. 624 p.

[48] Kaufman L, Rousseeuw PJ (1990) Finding groups in data: an introduction to cluster analysis. New York: John Wiley & Sons.

[49] Christensen HA, de Vasquez AM, Petersen JL (1999) Short report epidemiologic studies on cutaneous leishmaniasis in eastern Panama. Am J Trop Med Hyg 60: 54–57. 9988322 10.4269/ajtmh.1999.60.54

[50] Chao A, Colwell RK, Lin CW, Gotelli NJ (2009) Sufficient sampling for asymptotic minimum species richness estimators. Ecology 90: 1125–1133. 19449706 10.1890/07-2147.1

[51] Wheeler AS, Feliciangeli MD, Ward RD, Maingon RDC (1996) Comparison of sticky-traps and CDC light-traps for sampling phlebotomine sandflies entering houses in Venezuela. Med Vet Entomol 10: 295–298. 8887344 10.1111/j.1365-2915.1996.tb00747.x

[52] Galati EAB, Nunes VLB, Dorval MEC, Cristaldo G, Rocha HC, et al (2001) Attractiveness of black Shannon trap for phlebotomines. Mem Inst Oswaldo Cruz 96: 641–647. 11500761 10.1590/s0074-02762001000500008

[53] Faiman R, Cuno R, Warburg A (2009) Comparative efficacy of three suction traps for collecting phlebotomine sand flies (Diptera: Psychodidae) in open habitats. J Vector Ecol 34: 114–118. 20836811 10.1111/j.1948-7134.2009.00014.x

[54] Chaniotis BN (1983) Improved trapping of phlebotomine sand flies (Diptera: Psychodidae) in light traps supplemented with dry ice in a neotropical rain forest. J Med Entomol 20: 222–223. 6405036 10.1093/jmedent/20.2.222

[55] Alexander B (2000) Sampling methods for phlebotomine sandflies. Med Vet Entomol 14: 109–122. 10872855 10.1046/j.1365-2915.2000.00237.x

[56] Pugedo H, Barata RA, França-Silva JC, Silva JC, Dias ES (2005) HP: um modelo aprimorado de armadilha luminosa de sucção para a captura de pequenos insetos. Rev Soc Bras Med Trop 38: 70–72. 15717102 10.1590/s0037-86822005000100017

[57] Chaniotis BN, Neely JM, Correa MA, Tesh RB, Johnson KM (1971) Natural population dynamics of Phlebotomine Sandflies in Panama. J Med Entomol 8: 339–352. 4334027 10.1093/jmedent/8.4.339

[58] Alexander B, Maroli M (2003) Control of phlebotomine sandflies. Med Vet Entomol 17: 1–18. 12680919 10.1046/j.1365-2915.2003.00420.x

[59] Kirby MJ, Milligan PJ, Conway DJ, Lindsay SW (2008) Study protocol for a three-armed randomized controlled trial to assess whether house screening can reduce exposure to malaria vectors and reduce malaria transmission in The Gambia. Trials 9: 33. 18538004 10.1186/1745-6215-9-33 PMC2427015

[60] Lawyer PG, Young DG, Butler JF, Akin DE (1987) Development of *Leishmania mexicana* in *Lutzomyia diabolica* and *Lutzomyia shannoni* (Diptera: Psychodidae). J Med Entomol 24: 347–355. 3585930 10.1093/jmedent/24.3.347

[61] Feliciangeli MD, Reyes RM, Limongi JE (1988) Natural infection of *Lutzomyia ovallesi* (Diptera: Psychodidae) with parasites of the *Leishmania braziliensis* complex in a restricted focus of cutaneous Leishmaniasis in Northern Venezuela. Mem Inst Oswaldo Cruz 83: 393–394. 3271937 10.1590/s0074-02761988000300019

